# Photobiomodulation with simultaneous use of red and infrared light emitting diodes in the treatment of temporomandibular disorder: study protocol for a randomized, controlled and double-blind clinical trial

**DOI:** 10.1097/MD.0000000000014391

**Published:** 2019-02-08

**Authors:** Dowglas Fernando Magalhães de Sousa, Marcela Letícia Leal Gonçalves, Fabiano Politti, Renan Didier de Paula Lovisetto, Kristianne Porta Santos Fernandes, Sandra Kalil Bussadori, Raquel Agnelli Mesquita-Ferrari

**Affiliations:** Universidade Nove de Julho, São Paulo, Brazil.

**Keywords:** light emitting diode, photobiomodulation, range of mandibular movements, temporomandibular disorder

## Abstract

**Introduction:**

Temporomandibular disorder (TMD) is considered the main cause of orofacial pain of non-dental origin, and a public health problem. The symptomatology is muscular and/or articular pain, restriction of the mandibular range of motion, and changes in the mandibular movement pattern. Due to its complexity there are already treatments using various forms of therapy. Photobiomodulation using light sources, such as low-level laser or light emitting diodes (LED), with different wavelengths, in a single or combined form, allows one more therapeutic resource to be explored. The objective of this study is to evaluate the effects of photobiomodulation with the simultaneous use of red and infrared LEDs, on pain, range of mandibular movements, and on the electrical activity of masticatory muscles in individuals with TMD.

**Methods:**

A randomized, controlled, double-blind clinical trial is proposed, which will involve 33 individuals (n = 11 per group) of both sexes, ages 18 to 45 years in 3 groups: LED group; placebo group; and control group, submitted to 6 non-consecutive sessions of photobiomodulation totaling 2 weeks of treatment. The Research Diagnostic Criteria for Temporomandibular Disorders—RDC/TMD will be used to assess and determine the participants’ TMD. The pain will be assessed using the Visual Analog Scale – VAS, the mandibular range of motion will be determined with the aid of a digital caliper, and the electrical activity of the masticatory muscles will be verified by electromyography. A mixed plate of 18 red LEDs—660 nm and 18 infrared LEDs—850 nm with power of 3.5 mW per LED, 4.45 mW/cm^2^, radiant exposure of 5.35 J/cm^2^, will be used for photobiomodulation. The irradiated area will be 14.13 cm^2^, and energy of 75.6 J, in the TMJ region and in the bilateral masseter and temporal muscles. Participants from all groups will be reassessed after the first therapeutic intervention, and at the end of treatment.

**Discussion:**

We expect the use of photobiomodulation with LEDs, infra and red, to reduce pain, improve temporomandibular joint function in patients with TMD, and thus improve the general conditions of the patient.

## 1 Introduction

Temporomandibular dysfunction—TMD is a set of disorders that encompass the masticatory muscles, temporomandibular joints —TMJ, and the associated structures of the stomatognathic system, or all these structures compromising the system's functionality.^[1]^ It presents a multifactorial etiology^[2]^ and is considered the most common cause of orofacial pain of non-exclusively dental origin.^[3]^

The main symptom is muscular or joint pain, restriction of mandibular range of motion and changes in the pattern of movement of the mandible, but may present with tinnitus, vertigo, muscular incoordination, and biomechanical imbalance of the cervical region.^[4,5]^

Studies have reported that in the Brazilian population, 39.2% of patients had at least 1 sign or symptom of TMD.^[6]^ They occur in all age groups, mainly in adults, with a higher incidence in women in proportions ranging from 3:1.^[7,8]^ Hormones, such as estrogen, may be related to this predisposition.^[9]^ Women with TMD present twice the chance of a painful complaint,^[10]^ and it is already considered an important public health problem, occurring often for long periods and thus interfering with the daily activities of the individual.^[11]^

The complexity of TMDs encouraged the search for treatment of a multidisciplinary team and the use of different forms of therapy,^[12]^ among them, myorelaxant splints,^[13]^ massotherapy resources and mobilizations,^[14,15]^ as well as low level laser photobiomodulation.^[16,17]^ The low-level laser therapy (LLLT) is able to penetrate the tissues by influencing the synthesis, release and metabolism of substances involved in analgesia,^[18]^ stimulating blood flow, promoting anti-inflammatory action,^[19]^ and stands out for its easy application, with the minimum of contraindications and the possibility of shorter treatments.^[20]^ LLLT clinically reduces pain and improvement of mandibular movements.^[21]^

In addition to low-level laser photobiomodulation (LLLT), LED light presents as a further option in the treatment of TMD^[22,23]^ LEDs are semiconductor diodes (p–n junction), which when energized emit light,^[24]^ and present similar results to LLLT, with advantage of the cost of the device,^[25]^ the possibility of using clusters to irradiate larger areas at a single time, ^[26]^ the association of different wavelengths,^[22]^ and absence of side effects.^[27]^ Studies have suggested that LED therapy provides relief of pain, increase in range of motion, and improvement of muscular activity in patients with TMD.

The objective of the proposed study is to analyze the effects of photobiomodulation with simultaneous use in a single light emitting diode device—red LED (660 nm) and infrared LED (850 nm) on pain, amplitude of mandibular movements, and in electrical activity of masticatory muscles in individuals with temporomandibular dysfunction.

At the end of the study, we hope to find with the use of a cluster red and infrared LEDs, a reduction of pain in the masticatory muscles and in the region of the temporomandibular joint, improvement in temporomandibular joint function in TMD patients with the use of LED photobiomodulation and thus, improvement of the general conditions of the patient.

## 2 Materials and methods

### 2.1 Type of study

A controlled, randomized, double-blind clinical trial will be conducted at the dentistry and physiotherapy clinics of the University Nove de Julho—UNINOVE (Brazil) involving individuals with TMD distributed in 3 groups, including a placebo group and a control group. This protocol study was approved by the Committee of Ethics in Research with Human Beings of the University Nove de Julho (São Paulo, Brazil) under the number 2,962,857. All potential participants will receive clarification on the objectives and procedures, and those who agree to participate voluntarily will sign a declaration of free and informed consent, as stipulated in National Health Council resolutions 466/2012 and 510/2016.

**Figure 1:**
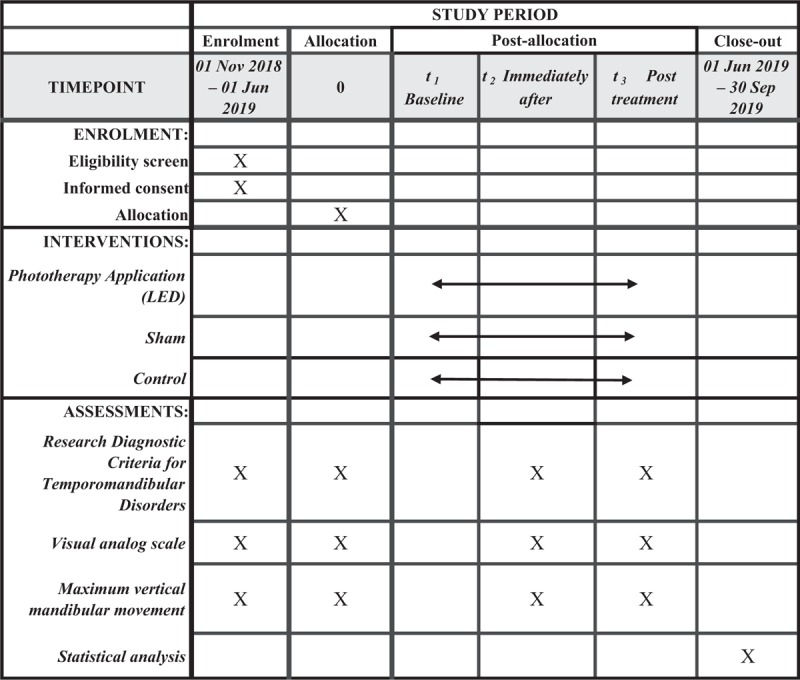
Standard Protocol Items: Recommendations for Interventional Trials (SPIRIT) figure as recommended by 2013 SPIRIT statement.

This protocol is in accordance with the 2013 SPIRIT (Standard Protocol Items: Recommendations for Interventional Trials) Statement. The SPIRIT Checklist can be found as an additional file and as Fig. 1. SPIRIT was developed to provide guidance in the form of a checklist of recommended items to include in a clinical trial protocol, to help improve its content and quality.^[28]^

### 2.2 Test record

Clinical record: clinicaltrials.gov as NCT 03696706, first published October 5, 2018; https://clinicaltrials.gov/ct2/show/NCT03696706?id=03696706&rank=1

### 2.3 Sample calculation

To calculate the sample size, data from the paper by Herpich et al^[22]^ was used. Initially we established an error = 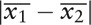, where 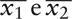 are the mean values of groups 1 and 2. Assuming that both samples have the same size (n_1_ = n_2_), the sample size can be obtained using the following relation: where 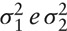 are the variances of groups 1 and 2, respectively. 
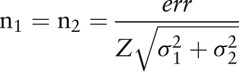


Assuming also that the studied groups have normal or approximately normal distribution, and that the sample size will be sufficiently large for a level of significance *α* = 0.05 a value of *Z* = 1.96 is obtained. For the sample size calculation it was also established that the power of the test should be 1 – *β* = 0.80. If the normal distribution hypothesis is rejected, the sample size should be corrected by approximately 5%. We obtain the following sample sizes: LED group: 11 participants, Placebo Group: 11 participants, and Control Group: 11 participants.

### 2.4 Recruitment and randomization

Volunteers from both sexes who will participate are students enrolled at University Nove de Julho in São Paulo, Brazil. The recruitment will be simple, since they will already be in the University.

Inclusion criteria: age between 18 and 45 years; clinical diagnosis of TMD, divided by the degrees of this dysfunction based on the Research Diagnostic Criteria for Temporomandibular Disorder—RDC/TMD (according to Table 1); have complete dentition (except third molars); mandibular deviation; and/or deflection.

**Table 1:**
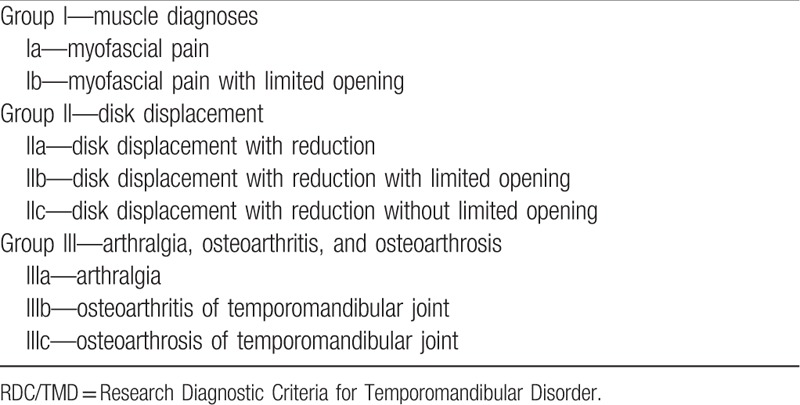
Diagnostic subgroups according to RDC/TMD.

Exclusion criteria: present occlusal alterations; use of any type of dental prosthesis; being in orthodontic or physiotherapeutic treatment; necessity of initiating the use of any type of medication during any of the phases of the study.

**Figure 2:**
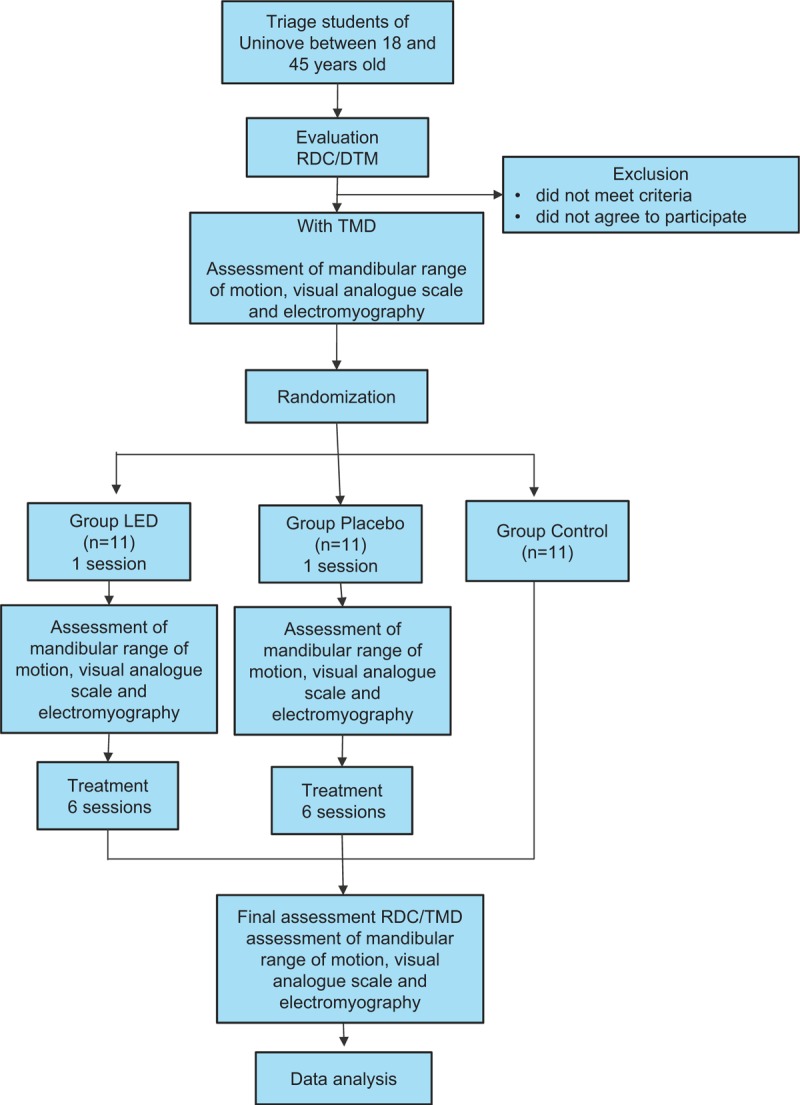
Flowchart activity.

The randomization will be performed, through the electronic address www.randomization.com, being a 1:1 randomization in blocks, with permutation and change in the size of the blocks. The participants will then be sent for the interventions according to each group, will be evaluated after the first therapeutic intervention, and all will be reevaluated at the end, following the same evaluation sequence used initially (Fig. 2).

## 3 Outcomes

### 3.1 Application of the LED therapy protocol

The sessions will be held in a reserved room, attached to the premises of the dental clinic, free of sound interference. The patient will remain seated, with the Frankfurt plane parallel to the ground. The LED plate shall be coated with clear PVC—disposable plastic for hygiene reasons and to avoid cross—contamination, and a previous facial cleaning with 70% alcohol of the irradiated site shall be performed.

The therapy will be performed with the Sportllux device (Cosmedical, Mauá, SP, Brazil), a plate containing 36 LED points in the regions of the temporomandibular joints, and in the regions of the masseter muscles and anterior portion of the temporal muscles, bilaterally, 3 times a week with interval between sessions, during 2 weeks, totalizing 6 treatment sessions. For the placebo group all measures described for the LED group will be adopted, however, the equipment will remain off. At the time of application only the volunteer to be treated and the researcher responsible will be present, both using specific eye protection. The LED apparatus is composed of a flexible rectangular plate (10 cm/12 cm), which adapts to the format of the area to be treated containing 18 red LEDs—660 nm and 18 infrared LEDs—850 nm, radiant exposure of 5.35 J/cm^2^, total power radiated by LEDs of 63 mW, irradiation of 4.45 mW/cm^2^ per point with an exposure time of 1200 seconds, resulting in an energy of 75 J per point, and total irradiated energy of 453.6 J per volunteer. The application will be in contact with the skin and the area of each beam of 0.7854 cm^2^. All parameters of the LED plates are shown in Table 2.

**Table 2:**
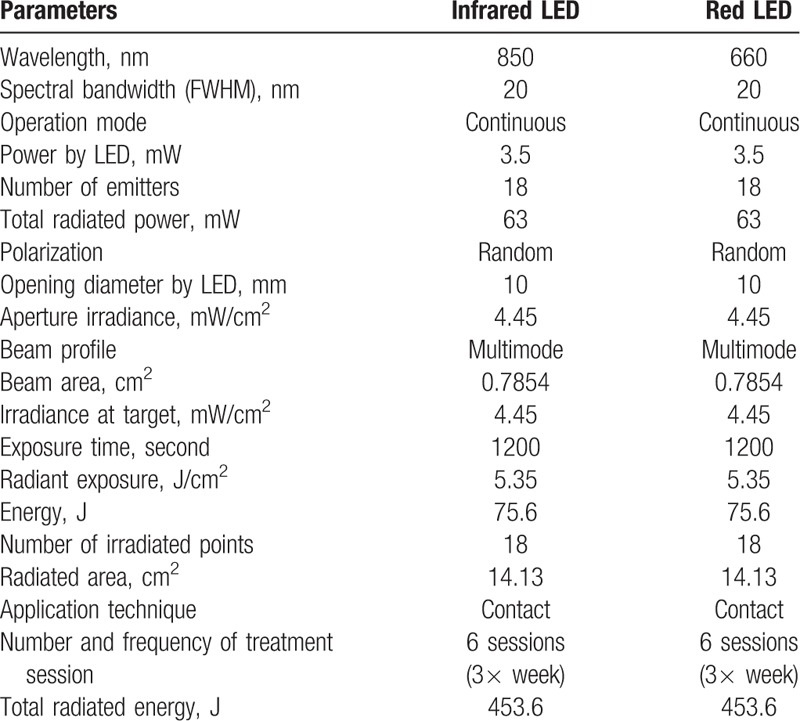
Parameters of the LED plate.

### 3.2 Research diagnostic criteria for temporomandibular disorder—RDC/TMD

This assessment tool is characterized as a biaxial instrument, composed of detailed physical evaluation of the pattern of buccal opening, vertical extension of mandibular movement, noises of TMJ on palpation for vertical movement extension, mandibular excursive movements, and TMJ noises upon palpation during lateral excursion and protrusion. The questionnaire consists of items involving health in general, oral health, history of facial pain, limitation of openness, noises, habits, bite, tinnitus, diseases in general, joint problems, headache, current behavior, economic and social profile.^[29]^

### 3.3 Visual analog scale

This scale will be used for pain assessment and consists of a 10 cm line with 0 (no pain) printed on one end and 10 (debilitating pain) printed on the other end. Participants will be asked to mark a spot on the line that represents the intensity of the current pain. The researcher will then use a ruler to record the distance of zero to obtain a numerical representation of the pain level. These procedures will be performed before and after the first session and at the end of treatment.

### 3.4 Mandibular range of motion

The volunteer will be instructed to open his mouth as much as possible. The maximum vertical mandibular movement will be measured (in mm) as the distance between the maxillary and mandibular central incisors determined with the aid of a digital caliper. The volunteer will then be instructed to put pressure on the lower teeth with the mouth open and move the jaw to the right and left to determine the excursion (distance between the upper and lower midpoints). These procedures will be performed before and after treatment. This procedure composes the clinical evaluation of RDC/TMD.

### 3.5 Recording of electromyographic signals

We will use a signal conditioning module with frequency bandpass filter between 20 and 1000 Hz, common mode rejection index >120 db. All data will be collected and processed using a 16-bit analog/digital converter with a sampling frequency of 2 kHz. Before placing the electrodes, the volunteer's skin will be cleaned with 70% alcohol to reduce the impedance,^[30]^ and the volunteer is asked to perform the trichotomy in the region if necessary. Active and disposable surface electrodes positioned in the area with the largest muscular volume of the masseter will be used, directed from the angle of the mandible to the lateral corners of the eyes, parallel to their fibers. In the same way, the anterior fibers of the temporal muscle will be palpated, and the electrodes will be positioned parallel to their fibers, bilaterally, performing muscular function.^[30]^ Participants will remain seated in a chair, with their backs fully supported by the backrest, Frankfurt plane parallel to the ground, eyes open, feet parallel and resting on the ground, and arms resting on the lower limbs.

The EMG signal will be captured in the following situations: postural position of mandibular rest; in non-habitual masticatory activity (isotonic) and; in isometry, that is, maximum voluntary contraction.^[31]^ In the isometry situation we will perform the evaluation with the interposition of parafilm and in the maximum usual intercuspation, and each situation will be repeated 3 times, with an interval of 2 minutes between each record for isotonia and isometry. In the situation of unusual chewing (isotonic) and in the isometry with interposition of parafilm, the volunteer will place it between the lower and upper premolars, first and second molars bilaterally. The Parafilme—M will be folded, leaving the size of approximately 35 mm, 15 mm wide and 3 mm thick, which obtained the lowest values of variability in electromyography records for masticatory activity, positioned bilaterally, or unilaterally between the inferior and superior premolars, first and second molars.^[32]^

### 3.6 Statistical analysis

The results obtained will be submitted to the Shapiro-Wilk statistical analysis to evaluate the normality. If the data obeys a Gaussian curve, the Analysis of Variance (ANOVA) of repetitive measures will be used with significance level *p* < 0.05. If the data are not parametric, the Kruskal–Wallis test will be used.

## 4 Discussion

Photobiomodulation, that is, the use of several light sources, sometimes in the same device, can be used as a therapeutic resource.^[33]^ A study of the histological analysis of rats showed positive results in the reduction of the carrageenan—induced inflammatory reaction in the TMJ after the use of the infrared LED—850 nm (100 mW, 10 J) and compared with the group using laser—780 nm (70 mW, 10 J). The criteria of histological evaluation were the percentage of neutrophils and lymphocytes in relation to the total of cells in the observed area, and the percentage of blood vessels in relation to the observed area.^[34]^ Prior to this, a study investigated the effects of phototherapy with the combination of different light sources on the nonspecific pain of a larger joint: the knee. The combination of 905 nm super-pulse laser and 875 and 640 nm LEDs were used, which proved effective in reducing pain and improving quality of life in patients with knee pain.^[35]^

The use of photobiomodulation with infrared LEDs 880 nm ± 20, energy density 4 J/cm^2^, presented results, after electromyographic evaluation, which pointed to the increase in electrical activity and muscle recruitment, this suggested the optimization of muscular activity with the use of therapy with LED, without causing side effects, such as elevation of strength and increase of blood lactate levels.^[27]^ Infrared LED phototherapy of 950 nm, but with different energy density and power, 3.2 J/cm^2^, 160 mW, had previously been used in skeletal muscles. The study did not present significant results when investigating its analgesic effect in pain induced experimentally in flexor muscles of the elbow of humans.^[36]^

Decreased pain and increased mandibular mobility in patients with TMD were also found after the use of LED photobiomodulation when applied to the masticatory muscles and points around TMJ. These results were achieved both with the use of 880 nm infrared LEDs, energy density of 7 J/cm^2^, and power of 0.03 W^[37]^; with 850 ± 10 nm, energy density 18 J/cm^2^, and power of 150 mW; as well as the use of red LED 630 ± 10 nm, 18 J/cm^2^, and power of 150 mW.^[23]^ The decrease in pain intensity in masticatory muscles was found even after single application, combining super-pulse laser 905 nm, 0.9 mW; Red LED—640 nm, 15 mW; infrared LEDs—875 nm, 15 mW, at different radiation doses: doses of 2.62 J/dot, 5.24 J/spot, and 7.86 J/spot in women with TMD.^[22]^

The effectiveness of photobiomodulation as in the treatment of TMD has been proven by several authors,^[17,22,37,38]^ but there are few controlled clinical studies analyzing the performance of photobiomodulation with different LED wavelengths in the same device with several simultaneous irradiation points in patients with TMD.

## 5 Statements

### 5.1 Methods of data collection

Four different authors, dentists, and physical therapists, who are qualified researchers in photobiomodulation therapy, and previously trained for data collection and evaluation, will perform the procedures. Each researcher will be responsible exclusively for each part of the study: the first researcher will be responsible for the screening and application of the questionnaires; the second will be responsible for randomization, concealment of allocation in groups (all other researchers will be blinded to the treatment of each volunteer), and application of photobiomodulation in all groups; the third one will be responsible for the execution of electromyography; and the fourth researcher will be responsible for the analysis and processing of the data. All data will be entered electronically, and the participants’ file will be stored in numerical order in a safe place and accessible only to the authors.

### 5.2 Interruption of interventions

The participant will be free to leave the study, at any time, without any kind of loss. If participants become ill or do not adapt to therapy, it will not be possible to continue therapy with LEDs. Interventions will be performed while the participants are in the university, to facilitate their participation and to avoid absences. No adverse effects are expected.

### 5.3 Availability of data and materials

The datasets generated and analyzed during the present study are available with the corresponding author when requested. After reviewing the data, the volunteers will be invited to a meeting and the results will be shared and the content will become public.

## Author contributions

**Conceptualization:** Dowglas Fernando Magalhães de Sousa, Sandra Kalil Bussadori, Raquel Agnelli Mesquita-Ferrari.

**Data curation:** Sandra Kalil Bussadori.

**Formal analysis:** Marcela Letícia Leal Gonçalves, Fabiano Politti.

**Investigation:** Dowglas Fernando Magalhães de Sousa, Marcela Letícia Leal Gonçalves, Fabiano Politti, Renan Didier de Paula Lovisetto.

**Methodology:** Dowglas Fernando Magalhães de Sousa, Marcela Letícia Leal Gonçalves, Fabiano Politti, Renan Didier de Paula Lovisetto, Sandra Kalil Bussadori, Raquel Agnelli Mesquita-Ferrari.

**Project administration:** Dowglas Fernando Magalhães de Sousa, Kristianne Porta Santos Fernandes, Sandra Kalil Bussadori, Raquel Agnelli Mesquita-Ferrari.

**Resources:** Kristianne Porta Santos Fernandes.

**Software:** Marcela Letícia Leal Gonçalves, Fabiano Politti, Renan Didier de Paula Lovisetto.

**Supervision:** Dowglas Fernando Magalhães de Sousa, Sandra Kalil Bussadori, Raquel Agnelli Mesquita-Ferrari.

**Validation:** Dowglas Fernando Magalhães de Sousa, Marcela Letícia Leal Gonçalves, Raquel Agnelli Mesquita-Ferrari.

**Visualization:** Dowglas Fernando Magalhães de Sousa, Marcela Letícia Leal Gonçalves, Fabiano Politti, Renan Didier de Paula Lovisetto, Kristianne Porta Santos Fernandes, Sandra Kalil Bussadori, Raquel Agnelli Mesquita-Ferrari.

**Writing – original draft:** Dowglas Fernando Magalhães de Sousa, Marcela Letícia Leal Gonçalves, Fabiano Politti, Renan Didier de Paula Lovisetto.

**Writing – review & editing:** Kristianne Porta Santos Fernandes, Sandra Kalil Bussadori, Raquel Agnelli Mesquita-Ferrari.
